# A route towards the fabrication of large-scale and high-quality perovskite films for optoelectronic devices

**DOI:** 10.1038/s41598-022-10790-z

**Published:** 2022-05-06

**Authors:** Ehsan Rezaee, Dimitar Kutsarov, Bowei Li, Jinxin Bi, S. Ravi P. Silva

**Affiliations:** Department of Electrical and Electronic Engineering, Advanced Technology Institute (ATI), University of Surrey, Guildford, Surrey GU2 7XH UK

**Keywords:** Devices for energy harvesting, Optical materials, Solar cells, Electronic devices, Energy

## Abstract

Halide perovskite materials have been extensively explored for their unique electrical, optical, magnetic, and catalytic properties. Most notably, solar cells based on perovskite thin films have improved their power conversion efficiency from 3.8% to over 25% during the last 12 years. However, it is still a challenge to develop a perovskite-based ink, suitable for upscaling the fabrication process of high-quality perovskite films with extreme purity, good crystallinity, and complete coverage over the deposition area. This is particularly important if the perovskite films are to be used for the scaled production of optoelectronic devices. Therefore, to make halide perovskites commercially available for various applications, it is vital to develop a reliable and highly robust deposition method, which can then be transferred to industry. Herein, the development of perovskite precursor inks suitable for use at low-temperature and vacuum-free solution-based deposition processes is reported. These inks can be further tailored according to the requirements of the deposition method, i.e., we propose their use with the industrially viable deposition technique called “slot-die coating”. Furthermore, a route for the preparation of low-cost and high-volume manufacturing of perovskite films on both rigid and flexible substrates is suggested in this paper. The presented approach is suitable for the fabrication of any functional layers of perovskites, that can be employed in various scaled applications, and it seeks the potential and the methodology for perovskite film deposition that is scalable to industrial standards.

## Introduction

Halide perovskite materials are depicted by the formula ABX_3_, where A and B are cations (A = caesium (Cs), methylammonium (MA), and/or formamidinium (FA); B = tin (Sn) and/or lead (Pb)), and X is an anion (chlorine (Cl), bromine (Br), and/or iodine (I))^1–5^. They offer unique combination of characteristics, such as solution processability, low material cost, great diversity, tunable band gap through interchanging cations, metals and halides in their structure, high carrier mobility, long carrier diffusion length, high absorption coefficients, low exciton binding energy, and long charge carrier lifetime^6–14^. Hence, perovskite materials are suitable for different of applications including thermo-electric devices, thin film resistors, capacitors, batteries, active catalysts for various reactions, lasing, light emission and detection, sensing, photovoltaics, and more^12,15–22^. Numerous studies are presently carried out for the realization of high-quality perovskite films for opto-electronic devices. However, the most exciting utilization of perovskite materials to date takes place when these materials are used in solar cells to effectively convert solar energy into electrical by showing power conversion efficiencies comparable or even superior to those of traditional silicon solar cells^23^. Coupling this to the long term potential of solar technology to provide cheap and green energy for the future makes a compelling case^1^. Furthermore, the fabrication of perovskite solar cells (PSCs) comes at a considerably lower cost compared to that of traditional silicon solar cells, due to a combination of low material cost and low energy needed for production. Therefore, it is not surprising that research communities and industry show significant attention to this new rival in solar cell technologies. The above-mentioned properties, applications, and achievements for perovskites highlight the importance of developing a manufacturing process for the preparation of large-scale perovskite films which can lead to the commercialisation of this technology.

However, most research to date reports on the fabrication of small laboratory-scale perovskite films, especially with the applications in optoelectronic devices in mind. Reported research outcomes are widely tied with the spin-coating deposition technique. Whilst spin-coating is an affordable fabrication method delivering reproducible results for small-area devices, it is not a scalable deposition process and does not allow for a continuous fabrication of functional layers as desired from an industrial point of view. The nature of the spin-coating process also leads to a high material loss, which makes it commercially and technically unrealistic for a large-scale industrial manufacturing of perovskite films. For large-area deposition, other solution-based but roll-to-roll (R2R) compatible techniques have been developed, including spray deposition, screen printing, blade coating, ink-jet printing, slot-die coating, and bar coating^24–34^. Among these, slot-die coating is considered as a particularly versatile technique, offering many advantages ranging from the capability of pre-metering of the coating process and results, and the ability of depositing a wide range of ink rheology; to an ink usage without any material loss, and the ability for high line speeds^35^. These advantages make slot-die coating a facile, suitable and transferable deposition technique from the laboratory environment to a high-throughput R2R fabrication of perovskite thin films in industry.

Employing the slot-die coating as the scalable deposition technique of choice, it is additionally required to develop suitable inks that contain solvent and precursor components compatible with the process requirements of this non-rotating deposition method. Furthermore, there is a fundamental difference in the formation kinetics of thin functional films depending on the deposition method used and the removal of remaining solvents from the perovskite layers. Whilst excess solvents are almost completely removed during the spin-coating, the pre-metered nature of the slot-die coating leads to the deposition of a wet film onto the substrate that can furthermore be dried by controlling factors such as temperature, pressure, and the intrinsic properties of the solvent mixture itself^36^. It is therefore not surprising, that there is no simple transfer of fabrication conditions between spin-coating and slot-die coating, as parameters of the deposition procedure will need to be tailored for a particular material precursor that is used during the deposition process. Studies have been carried out to bring in more compatible solvent systems to the perovskite precursor inks, as well as optimizing the ink concentration, to specifically match with the unique processing conditions of the scalable coating techniques^37^. Herein, uncontrolled nucleation and crystal growth procedure and ultimately, non-uniform perovskite films with poor quality and substrate coverage are formed when perovskite precursors based on commonly used high-boiling-point solvents with low vapour pressure remain long on the substrate^38^. By using solvents with a low boiling point, the quality of the obtained perovskite films was drastically improved over the last decade, leading to enhanced performance of PSCs and perovskite solar modules (PSMs)^39^.

Perovskite precursors are made of organic and inorganic materials with different solubility properties and affinities. A strong coordination bond is generally required to dissolve the inorganic precursor^40^. This is accomplished by using polar aprotic solvents such as dimethyl formamide (DMF), dimethyl sulfoxide (DMSO), γ-Butyrolactone (GBL) and N-Methyl-2-pyrrolidone (NMP)^39^. In this case, an extra thermal post-treatment step is inevitable to obtain perovskite films suitable for most optoelectronic applications. In contrast, rapid evaporation of the precursor solvent during the coating process can be used to achieve similar results and removes the additional annealing step. Herein, solvents with a low boiling point and a high vapour pressure can provide the required fast subtraction from the wet ink. However, they usually have low coordination ability, which makes it challenging to find a suitable solvent for inks compatible with scalable deposition processes. In 2019, Park et al. used 2-methoxyethanol (2ME) as the sole solvent for perovskite ink preparation^24^. Compared to DMF, as the most common solvent for spin coated inks, 2ME offers a lower boiling point (124 °C vs. 152 °C), and higher vapour pressure (6 vs. 2.7 torr). Additionally, 2ME can dissolve all the precursors used, providing a stable yellow ink, suitable for non-rotational coating techniques. Various 2ME-based perovskite inks were since developed and employed for different scalable coating techniques, such as D-bar coating, slot-die coating, blade coating, etc. resulting in high performance perovskite-based optoelectronic devices^41–43^.

In 2015, Zhou et al. found that methylammonium lead iodide (MAPbI_3_) can go through a reversible liquefaction process upon exposure to methyl amine (MA^0^) gas^44^. The following chemical reaction was suggested to take part in the MAPbI_3_ thin film:

1 $${\mathrm{CH}}_{3}{\mathrm{NH}}_{3}{\mathrm{PbI}}_{3}(\mathrm{s}) +\mathrm{ x}{\mathrm{CH}}_{3}{\mathrm{NH}}_{2}(\mathrm{g}) \rightleftarrows {\mathrm{CH}}_{3}{\mathrm{NH}}_{3}{\mathrm{PbI}}_{3}\bullet {\mathrm{xCH}}_{3}{NH}_{2}$$

Later, Zhang et al. have verified that MA gas exposure can effectively improve the surface morphology of the perovskite film, as well as its crystal structure with an induced preferred orientation, resulting in an enhanced charge transport ability of the film^45^. The established phenomenon has also been employed since for preparing various new perovskite precursor solutions, suitable for non-rotating thin film deposition techniques^38,46–48^. Acetonitrile-based MAPbI_3_ precursor inks were prepared by introducing MA gas to either a mixture of precursors in the solvent, or directly onto pure MAPbI_3_ single crystal and/or powder, followed by adding the solvent to the obtained viscous liquid. The obtained stable ink was shown to be a suitable choice for a large-scale deposition process, offering a rapid solvent removal and therefore, a quick drying process^49,50^. To achieve a higher control over the nucleation and crystal growth during the film drying, techniques such as air/nitrogen blowing, and heated substrate, along with using a variety of additives and post-treatments have been developed so far^51,52^.

In this study, we aim to establish a reliable and reproducible procedure for perovskite thin film preparation over large areas. Wise selection of the coating method and parameters, compatible with a stable and well-suited perovskite ink, led to the formation of perovskite thin films with micrometer sized grains. The obtained film offered a full coverage over the deposited area, with a smooth surface profile, which is preferable for the deposition of subsequent films in the device. An optimized nitrogen gas blowing during the slot-die coating of the perovskite thin film helped to accelerate the nucleation step and resulted in a high level of crystal growth. The deposition process reported in this paper is carried out at room temperature and is suitable for the fabrication of large perovskite crystal grains over large areas on rigid and flexible substrates. This can lead to a simple but efficient coating procedure of perovskite films which offers a cost-effective manufacturing of perovskite layers, compatible with various applications in optoelectronic devices.

## Materials and methods

### Materials

Pre-patterned ITO coated glass substrates had dimensions of 78 mm × 78 mm × 0.7 mm. Each substrate had six ITO stripes with 10 mm width, and 2 mm etched area between two adjacent stripes. Lead diiodide (PbI_2_, 99.99%) was purchased from TCI (Tokyo Chemical Industry). Methylammonium iodide (MAI) was purchased from GreatCell Solar Materials. Methylamine solution (40 wt% in H_2_O) was purchased from Merck. Drierite column was purchased form Cole-Parmer. Acetonitrile (ACN), dimethylformamide (DMF) and N-Methyl-2-pyrrolidone (NMP) were anhydrous grade and purchased from Sigma-Aldrich. Poly[*N*,*N′*-bis(4-butylphenyl)-*N*,*N′*-bis(phenyl)benzidine] (poly-TPD, M_w_ = 85 k) and poly[(9,9-bis(3′-((N,N-dimethyl)-N-ethylammonium)-propyl)-2,7-fluorene)-*alt*-2,7-(9,9-di-octylfluorene)] dibromide (PFN-Br or called PFN-P2) were purchased from 1-Material. 2,3,5,6-Tetrafluoro-7,7,8,8-tetracyanoquinodimethane (F4TCNQ) was purchased from Xi’an Polymer Light Technology Corp. [6, 6]-phenyl-C_61_-butyric acid methyl ester (PC_61_BM, 99.5%) was purchased from Solenne. Bathocuproin (BCP, 98%) was purchased from Alfa Aesar. Toluene (99.85%) and chlorobenzene (CB, 99.8%) were purchased from Acros Organics. All chemicals were used as received.

### Ink preparation

Pure ACN-based perovskite ink was prepared by mixing 1 mmol of MAI and 1 mmol of PbI_2_ in a small vial with 1 mL of ACN. 40 mL of methylamine solution in H_2_O was loaded in a gas washing bottle. The methylamine solution was then bubbled under ambient conditions with dry nitrogen at 0.8 LPM (litre per minute) and passed through a 500 g Drierite column and into the perovskite precursor solution. All connections were made with Tygon tubing. During the bubbling, the perovskite precursor mixture was under mild shaking. The solids in the precursor mixture would go from deep black, over dull grey, to a clear yellow solution after about 2 min. The same procedure was used for preparing an ACN/NMP-based perovskite ink. The precursor mixture was prepared by adding 1.1 mmol of MAI and 1.1 mmol of PbI_2_ to 894 µL of ACN and 104 µL of NMP, respectively. The DMF based solution was prepared by mixing 1.1 mmol of MAI and 1.1 mmol of PbI_2_ to 894 µL of DMF and 104 µL of NMP, respectively. The clear yellow ink was obtained after few minutes of mild shaking. The obtained inks were filtered through 0.20 μm PTFE microfilter before use.

### Thin film fabrication methods

To study the proposed coating procedure and the quality of the perovskite thin films, samples with the structure of glass/ITO/MAPbI_3_ were fabricated. Slot-die coated samples were prepared on pre-patterned ITO coated glass substrates (15 Ω/sq). The gap between the slot-die head and the substrate was set by using micrometer adjustment screws and feeler gauges. An air knife (Exair Standard Air Knife) was positioned at the downstream side of the coating head with a distance of 100 mm from the slot-die head and a hight of 25 mm from the substrate. Compressed nitrogen was used as the feed gas. Substrates were cleaned using ultrasonic bath with Helmanex III detergent, deionized water, acetone, and isopropanol (IPA) sequentially, followed by blow drying with compressed nitrogen. For coating the perovskite thin film, a slot-die head, fitted with 100 μm thick shim and a meniscus guide with 1000 μm tab length was used, with a 720 mm coating width. A coating speed of 10 mm s^−1^ was used, with an ink flow rate of 120 μL min^−1^. Films were annealed at 100 °C for 20 min in air.

### Device fabrication

The detailed fabrication process of *p-i-n* devices is referenced by previous reports from our group^53,54^. Pre-patterned ITO substrates (20 × 20 mm^2^) were sonicated with detergent, deionized water, acetone, IPA and cleaned by an O_2_ plasma asher (10 min). Then, all substrates were transferred into a N_2_-filled glove box. The poly-TPD solution (1 mg mL^−1^ in toluene with 0.2 mg mL^−1^ F4TCNQ) was spin-coated at a speed of 2000 rpm (with a speed ramp of 2000 rpm s^−1^) for 30 s and then annealed at 130 °C for 10 min. To improve the wettability, 0.4 mg mL^−1^ PFN-Br was spin-coated at 5000 rpm (with a speed ramp of 2500 rpm s^−1^) for 30 s. Perovskite films were deposited using the slot-die coating process described above. A small amount of DMSO (24 mol%) was added to the pure ACN-based ink for PSC fabrication. Subsequently, the samples were annealed in air at 100 °C for 20 min on a hotplate in a fume hood. Then, as-crystalized perovskite films were transferred into a N_2_-filled glove box. 30 mg mL^−1^ PC_61_BM solution (in CB) and 0.5 mg mL^−1^ BCP solution (in IPA) were spin-coated at 2500 rpm (30 s) and 5000 rpm (25 s), respectively. Finally, all substrates were transferred outside the glove box for the electrode deposition. 100 nm of Ag film was thermally evaporated as a counter electrode using a shadow mask and the device areas were around 0.25 cm^2^.

### Device characteristics

The X-ray diffraction crystallographic data for the samples was collected using a PANalytical X'Pert Pro powder diffractometer in a Bragg–Brentano geometry using a Cu Kα target (45 kV, 30 mA). Scanning Electron Microscope (SEM) images were taken using a Jeol JSM-7800F field emission gun electron microscope, adjusting the working distance around 10 mm and using an acceleration voltage of 5 kV and a probe current of the order of 9 μA. Atomic force microscopic (AFM) images were obtained with a Bruker Dimension Edge in a tapping mode. The film thickness was measured by a surface profilometer (Dektak XT). PL was measured by HORIBA Xplora Plus micro Raman Spectrometer. The time-resolved photoluminescence (TRPL) was obtained using Picoquant FluoTime 300 fluorescence Spectrometer. The decay curves were simulated according to the following formula

2 $${\tau }_{avg}=\left({A}_{1}{\tau }_{1}^{2}+{A}_{2}{\tau }_{2}^{2}\right)/\left({A}_{1}{\tau }_{1}+{A}_{2}{\tau }_{2}\right)$$

The *J–V* measurements of the PSCs were performed in air and at room temperature without any encapsulation. The simulated light was one-sun AM 1.5G illumination (100 mW cm^−2^) delivered by a solar simulator (Enlitech, SS-F5-3A). The light intensity was calibrated by using a standard monocrystalline silicon solar cell with a KG-5 filter same as previously reported^55^, and a reference cell purchased from Fraunhofer ISE CalLab (ISE001/013-2018). All devices were measured by Keysight B2901A source meter both in the reverse scan (1.2 →  − 0.2 V, step 0.02 V) and forward scan (− 0.2 → 1.2 V, step 0.02 V). To ensure accuracy, a mask with an aperture area of 0.09 cm^2^ was employed during the measuring process.

## Results and discussion

**Figure 1:**
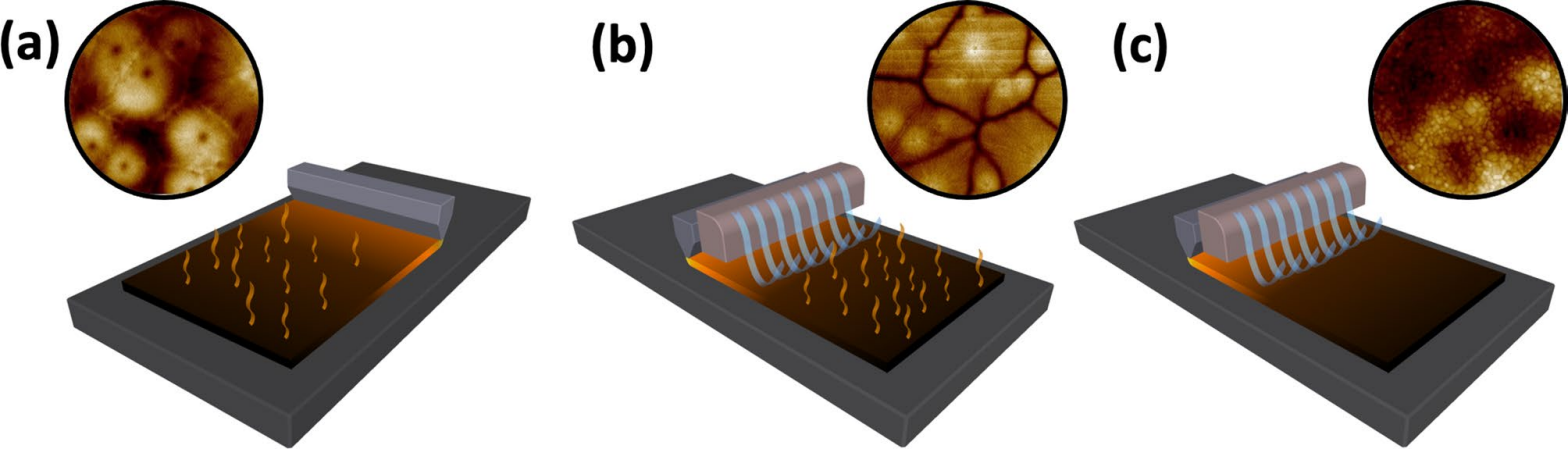
Schematic representation of different coating conditions used in this study: (**a**) coating at elevated temperature without N_2_ gas blowing; (**b**) coating at elevated temperature with N_2_ gas blowing; and (**c**) coating at room temperature with N_2_ gas blowing.

For the perovskite ink, acetonitrile (ACN; boiling point of 82 °C) and dimethyl formamide (DMF; boiling point of 153 °C) were chosen as solvents with low and high boiling points, respectively, as well as N-Methyl-2-pyrrolidone (NMP) as the coordinative additive. The three different solvent systems used for preparing the perovskite inks are as follow: (1) pure ACN; (2) ACN as the primary solvent and NMP as a coordinating co-solvent (ACN/NMP; 8.4:1 volume ratio of ACN:NMP); and (3) DMF as the primary solvent with NMP as a co-solvent (DMF/NMP; 8.4:1 volume ratio of DMF:NMP). Equimolar amounts of MAI and PbI_2_ were added to the precursor solutions, delivering the concentrations of 1, 1.1 and 1.1 M, for ACN, ACN/NMP, and DMF/NMP inks, respectively. In case of ACN-based solutions, the clear yellow MAPbI_3_ inks were only obtained after blowing dry methylamine (MA) gas into the precursor mixtures. The prepared inks were coated on large-area glass/ITO substrates using the slot-die coating technique. To improve the drying step and to achieve a better control over the nucleation and crystal growth process, treatments such as variation of the substrate temperature and the use of a N_2_ air knife were introduced during the slot-die coating step of the perovskite ink. A schematic representation of the coating conditions is shown in Fig. 1 and the coating parameters for each ink are listed in Table 1.

**Table 1:** Parameters used for slot-die coating of perovskite inks.

Sample code	Ink	Coating speed (mm s^-1^)	Ink flow rate (μL min^-1^)	Gap between slot-die head and substrate (µm)	Substrate temperature (°C)	N_2_ blowing flow pressure (bar)	Shim width (mm)	Shim thickness (µm)	Treatment
1	ACN	10	120	200	60	0	72	100	–
2	ACN	10	120	200	60	2	72	100	–
3	ACN	10	120	200	60	2	72	100	MA-healing
4	ACN	10	120	200	R.T	2	72	100	–
5	ACN/NMP	10	120	200	R.T	0	72	100	Anti-solvent
6	DMF/NMP	10	120	200	R.T	0	72	100	Anti-solvent

**Figure 2:**
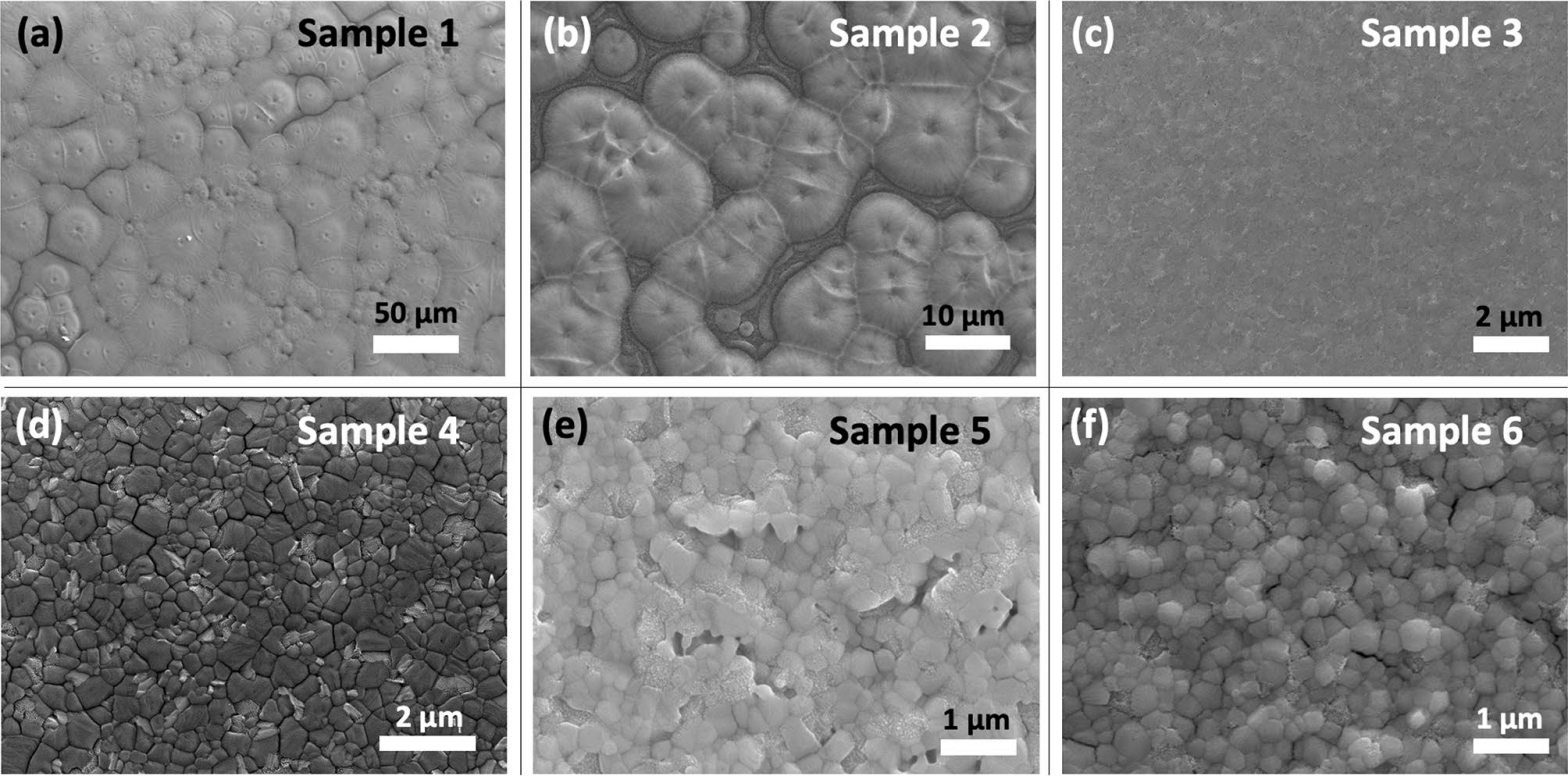
SEM images of MAPbI_3_ thin films deposited on ITO substrates using the following methods: (**a**) ACN-based ink deposited on a hot substrate (sample 1); (**b**) ACN-based ink deposited on a hot substrate with N_2_ blowing (sample 2); (**c**) ACN-based ink deposited on a hot substrate with N_2_ blowing and MA-induced post-treatment (sample 3); (**d**) ACN-based ink deposited at room temperature with N_2_ blowing (sample 4); (**e**) ACN/NMP-based ink deposited at room temperature with an anti-solvent treatment (sample 5); (**f**) DMF/NMP-based ink deposited at room temperature with an anti-solvent treatment (sample 6).

**Figure 3:**
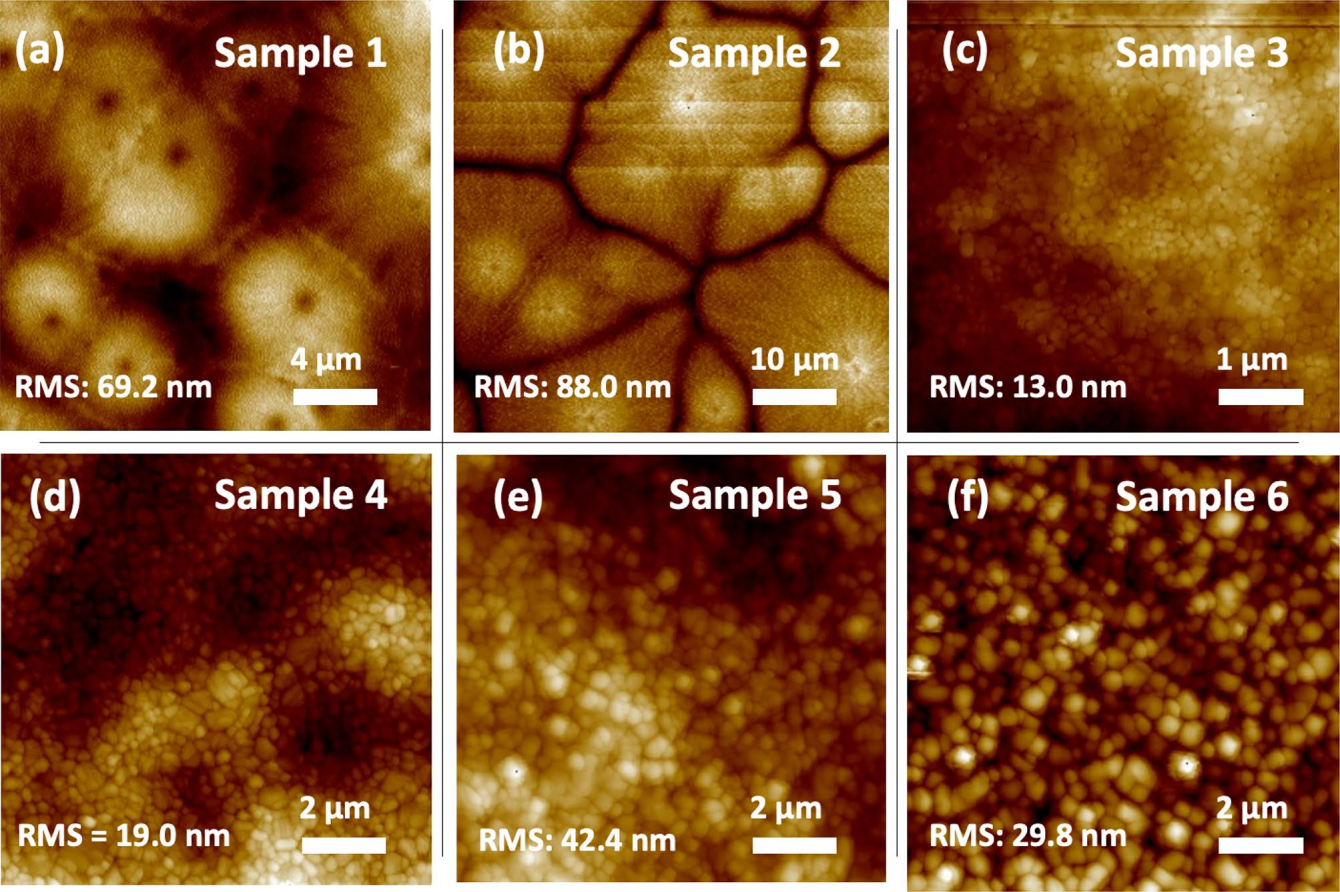
AFM images of MAPbI_3_ thin films deposited on ITO substrates using the following methods: (**a**) ACN-based ink deposited on a hot substrate (sample 1); (**b**) ACN-based ink deposited on a hot substrate with N_2_ blowing (sample 2); (**c**) ACN-based ink deposited on a hot substrate with N_2_ blowing and MA-induced post-treatment (sample 3); (**d**) ACN-based ink deposited at room temperature with N_2_ blowing (sample 4); (**e**) ACN/NMP-based ink deposited at room temperature with an anti-solvent treatment (sample 5); (**f**) DMF/NMP-based ink deposited at room temperature with an anti-solvent treatment (sample 6).

The temperature of the substrate, along with gas blowing over the wet ink through an air knife during the slot-die coating process are known to significantly affect the film formation mechanism and therefore, the crystallization and the final quality of the perovskite film^56^. A heated substrate during the coating can result in crystal-growth-driven (CGD) type of perovskite film, in which a lower degree of supersaturation and less nucleation sites occurs in the wet film during the drying step due to the delayed nucleation step. This eventually leads to large grain clusters in the film, whose quality being controlled by the crystal growth step^56^. Initially, to achieve a CGD-type MAPbI_3_ film, a substrate temperature of 60 ˚C was chosen, due the low boiling point and high vapour pressure of ACN (the coating condition is shown in Fig. 1a). Sample 1 in Table 1 represents the slot-die coated perovskite film onto a heated substrate, using pure ACN-based MAPbI_3_ ink. As shown in Fig. 2a, the SEM image of the sample 1 demonstrates the formation of low amount of nucleation sites in the film during the drying step, resulting in grain clusters as large as 50 µm and consequently a partial coverage of the film over the deposited area. Slot-die coating the pure ACN-based ink on heated substrates results in high surface roughness for the dried film, as it is observed in the AFM image of the sample 1 (Fig. 3a). Such topology of the perovskite film can negatively impact the adhesion properties and formation quality of any subsequent layers inside the device.

**Figure 4:**
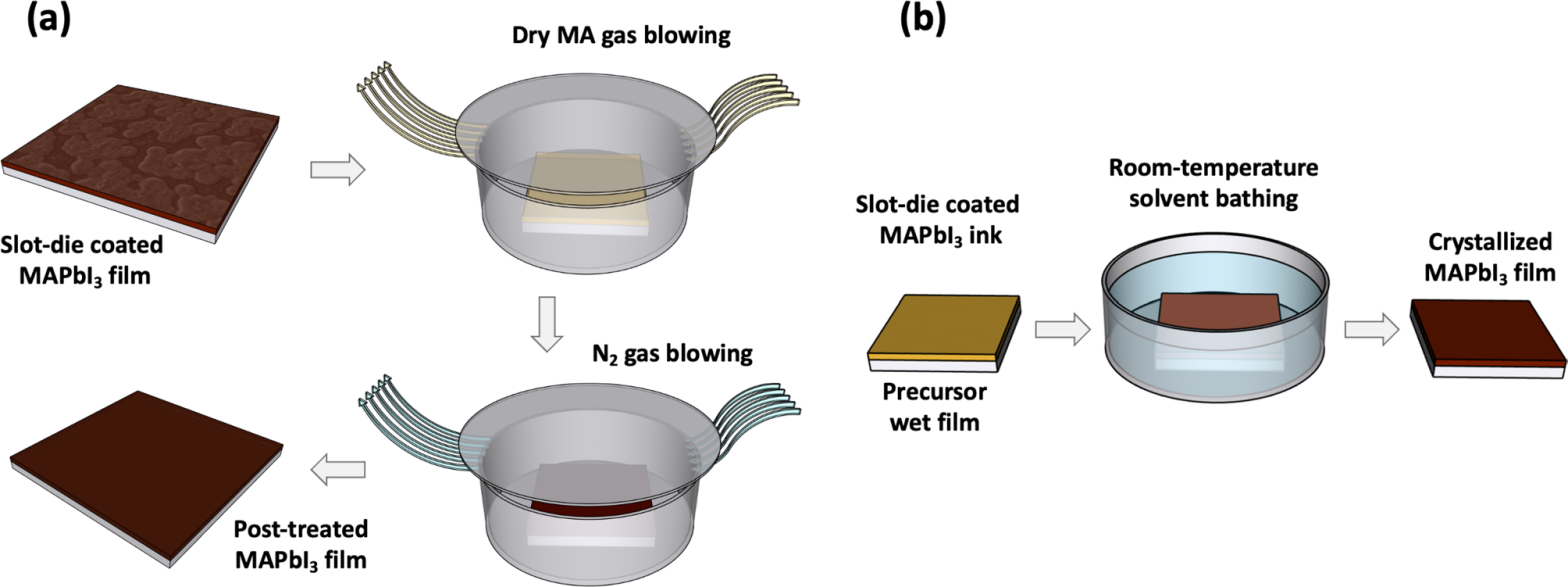
Schematic illustration showing: (**a**) MA-induced healing post-treatment performed on dry MAPbI_3_ perovskite thin film and (**b**) anti-solvent dipping post-treatment on wet MAPbI_3_ perovskite film.

As an alternative to the commonly used anti-solvent approach during spin-coating, gas blowing is considered as an effective method for the solvent removal in non-rotational scalable deposition techniques. It facilitates a fast increase in the degree of supersaturation for the wet film during the drying process and leads to a rapid nucleation step and consequently, a nucleation-driven (ND) type of perovskite film growth. The ND film formation mechanism, usually results in densely packed small crystal grains in the final perovskite film, ensuring high quality and smooth film, with a full coverage over the deposited area^56^. Therefore, in the next step, we combined the substrate heating approach with N_2_ gas blowing during the slot-die coating process in order to examine the impact of the additional solvent removal technique on the crystallization mechanism of the perovskite layer. As shown in Fig. 1b, an air knife was attached behind the slot-die head, and it was used to introduce a high-pressure gas blowing to the deposition process. Interestingly, the film formation mechanism went through the CGD path, resulting in large grain clusters forming around the nucleation sites during the drying step of the film deposition. Regarding the surface SEM image of sample 2 presented in Fig. 2b, the film quality slightly drops by introducing the gas blowing to the deposition process. It is known that in order to achieve a high-quality perovskite film, a supersaturation state should be reached very fast after ink deposition. The supersaturation state can be achieved through either a solvent extraction technique or solvent evaporation process. A fast supersaturation is usually obtained with a proper solvent extraction at the right time after ink deposition, as in the very common anti-solvent treatment for spin-coating process. During the preparation of sample 2, the gas blowing could induce the nucleation step as early as possible. However, with rapid evaporation of ACN due to the substrate temperature, the crystal growth was still the main driving force for the film formation procedure. Therefore, the drying process of the film was completed before accomplishing a high amount of nucleation sites in the wet film. Using the gas blowing method in combination with a hot substrate caused even lower coverage for the film compared to sample 1, as the crystal grain clusters now had less time to grow, resulting in huge gaps between the clusters. This is furthermore confirmed by the AFM images of the films in Fig. 3b, where in comparison to sample 1 (with the RMS value of 69.2 nm), a higher surface roughness (88.0 nm), and a lower surface coverage with clear gaps between the grain cluster can be observed in the sample 2. We believe that such a coating approach will only be suitable for solvent systems where the primary solvent component has a high boiling point, which offers more tolerance for an elevated temperature during coating. We suggest that perovskite films similar to sample 2 can only be used if an additional post-treatment, that can offer reformation in crystal grain order and packing inside the film, are applied during the device fabrication. To explore this idea, we chose to use a methylamine (MA)-induced healing step to investigate the effect of this post-treatment procedure on the low-quality perovskite film of sample 2 (a schematic illustration of the post-treatment is shown in Fig. 4a). Indeed, a significant improvement of the quality of the perovskite film originating from sample 2 can be observed in sample 3. The ND-type film was only achieved after post-treatment of the as-prepared MAPbI_3_ film through introducing dry MA gas onto the films for few seconds. During this process, the MAPbI_3_ film bleaches completely after the exposure to the dry MA gas, going through a pseudo-liquefaction process and recrystallized immediately after removing the MA gas^44^. The post-treated MAPbI_3_ layer converted from the rough and non-homogeneous sample 2 to a smooth and mirror-like film in sample 3, which shows a higher overall quality and better coverage over the large area, as evident from the SEM image in Fig. 2c. Comparing the AFM images of the two samples confirms the enhanced quality of the film due to the post-treatment, leading to a very smooth thin film with small crystal grains densely packed together (Fig. 3c). However, we found that the reproducibility of this approach is low, which makes it unsuitable for a large-scale manufacturing environment, where high production yields are needed. Additionally, introducing an extra step to the fabrication process can compromise the simplicity of the production path and make it less desirable for industrial application.

To this end, we returned to room temperature coating to have a better control over the film formation mechanism. Sample 4 was thus prepared at room temperature and accompanied by a N_2_ gas blowing (Fig. 1c). Interestingly, ND-type film formation mechanism was dominant when an efficient solvent removal approach was introduced to the deposition procedure by blowing the N_2_ gas with a pressure of 2.0 bar. A significant improvement was seen in the quality of the perovskite film through changing the film formation mechanism. As shown in the SEM image in Fig. 2d and in the cross-section SEM image in Suppl Fig. S1, crystal grains with sizes up to a micrometer were formed inside the film, tightly packed to deliver a smooth perovskite film. In addition, the surface morphology of the perovskite film in Fig. 3d shows an RMS value of 19 nm, which indicates a very smooth film topology. These results point out, that room temperature coating of an ACN-based perovskite ink allows for inducing a nucleation step through gas blowing, which must be carried out at the right time before the solvent evaporates due to the energy coming from the temperature of the environment. Therefore, it is extremely important to remove the solvent in a rapid step. Only in that case one can expect the formation of films comparable with those employed in spin-coated high-performance devices. The developed procedure is furthermore highly reliable and delivers high quality perovskite films with high reproducibility as shown in Suppl Fig. S2. We also gradually increased the slot-die coating speed from 4.2 to 18 mm s^−1^ and similar high-quality films were obtained each time, indicating the capacity of the developed perovskite ink and deposition procedure to fabricate perovskite layers in raised speeds (Suppl Fig. S3). Additionally, we applied the developed method for MAPbI_3_ ink fabrication on FAPbI_3_- and MA_0.3_FA_0.7_PbI_3_-based inks and successfully achieved inks suitable for large-scale deposition.

To compare the results of the developed coating method with the anti-solvent treated perovskite films, we prepared samples 5 and 6 (the schematics of the anti-solvent dipping post-treatment is shown in Fig. 4b). The anti-solvent dipping method can be considered as a promising alternative to the introducing of an anti-solvent during the spin-coating procedure. Although the anti-solvent dipping technique offers the compatibility to the scalable deposition approaches, a large processing window for the solvent–solvent extraction is highly desirable for achieving a successful fabrication process of high-quality perovskite films^57^. We chose diethyl ether (DEE) as an anti-solvent in this study, providing long application time, as the main requirement for the reproducibility of high-quality perovskite films^58^. To further increase the processing window and develop a fabrication process suitable for industrial applications, high boiling point solvents such as NMP can be added to the ink as the coordinating solvent^59^. In our study, NMP was added to the ACN-based MAPbI_3_ ink (equimolar to MAPbI_3_) to suppress the fast drying and crystal growth inside the wet film. Using the ACN/NMP ink, a room temperature slot-die coating without N_2_ gas blowing allows for the anti-solvent dipping technique to be performed onto the as-prepared wet films (sample 5). Similarly, using the solvent engineering technique for DMF, one can achieve a better miscibility into the selected anti-solvent and therefore, a faster nucleation step for the film formation. Thus, with sample 6 we also explored the impact of changing the primary solvent in the ink from ACN to DMF for the anti-solvent dipping method and investigated the final quality of the films. Considering the SEM images of sample 5 and 6 in Fig. 2e and f, in both cases, the perovskite film was clearly formed through the ND-type mechanism, resulting in densely packed crystal grains offering a fully covered thin film over the coating area. This approves a successful solvent removal during the anti-solvent dipping step, which can guarantee a good quality of the perovskite film. However, the DMF-based ink led to the formation of larger grain sizes of the MAPbI_3_ crystals. With its higher miscibility with diethyl ether as the anti-solvent, DMF could be removed more efficiently than ACN during the anti-solvent dipping step, offering a superior nucleation step, and consequently a smoother layer. AFM images presented in Fig. 3e and f also suggested a lower surface roughness of the DMF-based film compared to the ACN-based alternative. Comparing the crystal grain size of the DMF-based MAPbI_3_ film with that of sample 4 indicates that the largest grains, along with the best quality for the MAPbI_3_ layer can be achieved through our optimized room temperature, fast, simple, and low-cost coating method. Therefore, with an optimized ink formulation and suitable anti-solvent selection, the most favoured solvent engineering technique in the small area perovskite film formation can be transferred to large-area device fabrication procedures. However, a wide processing window is required to achieve high quality perovskite thin film through the anti-solvent dipping method. Otherwise, an early or late introduction of the anti-solvent, which can be the case during the moving substrates from the coating stage into the anti-solvent bath, will results in either a film full of pinholes and spiral patterns, or a film with a rough surface^57^. While the anti-solvent dipping method can guarantee an effective nucleation step, the gas blowing technique can offer a faster process with much higher reproducibility.

**Figure 5:**
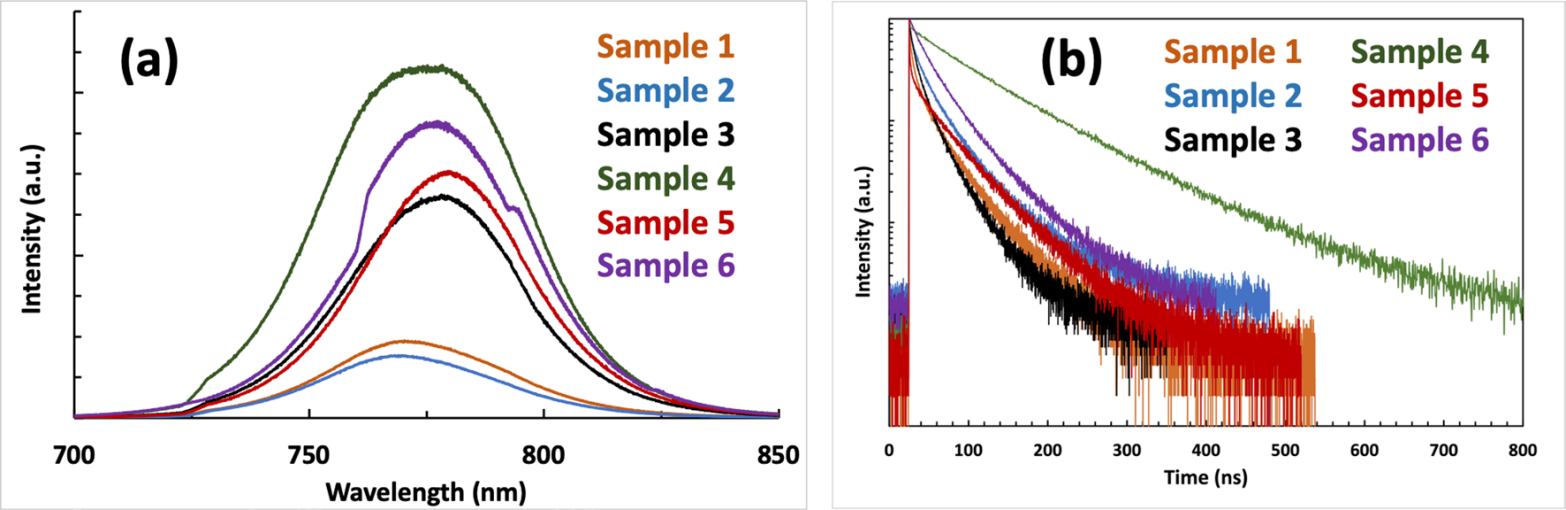
Spectra of: (**a**) PL; and (**b**) TRPL of the prepared perovskite thin films for samples 1–6.

The steady-state PL spectroscopy was used to further investigate the optoelectronic properties of the deposited perovskite thin films of samples 1 to 6. As shown in Fig. 5a, samples 1 and 2 showed the lowest PL intensity, indicating the poor quality of the formed perovskite thin film with the partial coverage of the film over the deposited area, which is in accordance with the results obtained from the imaging characterization techniques. Comparing the PL spectrum of sample 2 with the one for sample 3 confirms the effectiveness of the MA-induced healing post-treatment in passivating the defects inside the perovskite layer through altering the film formation mechanism from CGD-type to ND-type. This resulted in a more uniform crystal grain packing and a large reduction in non-radiative pathways. The highest PL intensity was observed for sample 4. This strongly approves the efficiency of the developed film fabrication recipe in delivering the highest quality for the perovskite layer with the lowest defects inside the film. Compared to the recipe for preparing sample 3, the deposition path for sample 4 offers more controlled fabrication conditions with less steps, leading not only to a higher reproducibility for the whole process, but also a more effective defect passivation for the final film. Both samples 5 and 6 displayed a relatively high PL intensity in their spectra, suggesting an overall decent optoelectronic performance of the achieved thin films, which can be associated with the advantage of the ND-type over CGD-type film formation mechanism in producing packed perovskite grains inside the bulk film. The TRPL spectra of the prepared samples are presented in Fig. 5b. The decay curves are fitted using Eq. (3), and the main fitted data is displayed in Suppl Table S1.

3 $$y\left( t \right) = y_{0} + A_{1} e^{{\left( { - t/\tau _{1} } \right)}} + A_{2} e^{{\left( { - t/\tau _{2} } \right)}}$$

As expected, the calculated average carrier lifetime of 98.49 ns for sample 4 was considerably higher than those of other samples, which is consistent with its superior film quality and coverage, as well as its lowest defects density. Sample 2 showed the lowest carrier lifetime of 21.87 ns, which was slightly improved through MA-induced healing post-treatment (sample 3), reaching to the value of 30.14 ns. As it was mentioned, the MA-induced healing technique could favourably alter the film formation mechanism from CGD- to ND-type however, the PL and TRPL results confirmed that it also can results in films with a high level of defect density. With its higher film coverage compared to sample 2, and yet CGD-type film, sample 1 showed a slight increase in the average lifetime, reaching to 34.05 ns. Finally, the two anti-solvent post-treated samples 5 and 6 showed higher lifetimes (38.36 and 43.34 ns, respectively), proving the advantage of ND- over CGD-type perovskite films.

Crystal structure of the perovskite thin films of samples 1 to 6 were analysed by X-ray diffraction (XRD) (shown in Suppl Fig. S4). The positions of the peaks were measured and labelled with the corresponding Miller index^60^. The presence of multiple peaks, including (110), (114), (121), and (312) in the spectra of the samples 1, 2, 5 and 6 suggests the obtained films did not feature a preferred orientation. Additionally, these samples showed a considerably lower peak intensity in comparison to those of the samples 3 and 4. It strongly confirms the higher crystallinity of the perovskite thin films for the samples 3 and 4. A significantly stronger (110) and (220) peaks over other peaks in the samples 3 and 4, demonstrates the dominancy of (110) plane over other crystal plane orientation in these samples, and indicates an enhancement in the preferred crystal orientation in the <110> direction. Furthermore, the observed narrower (110) and (220) peaks in sample 4, compared with those of sample 3, determines the higher level of crystallinity in sample 4.

**Figure 6:**
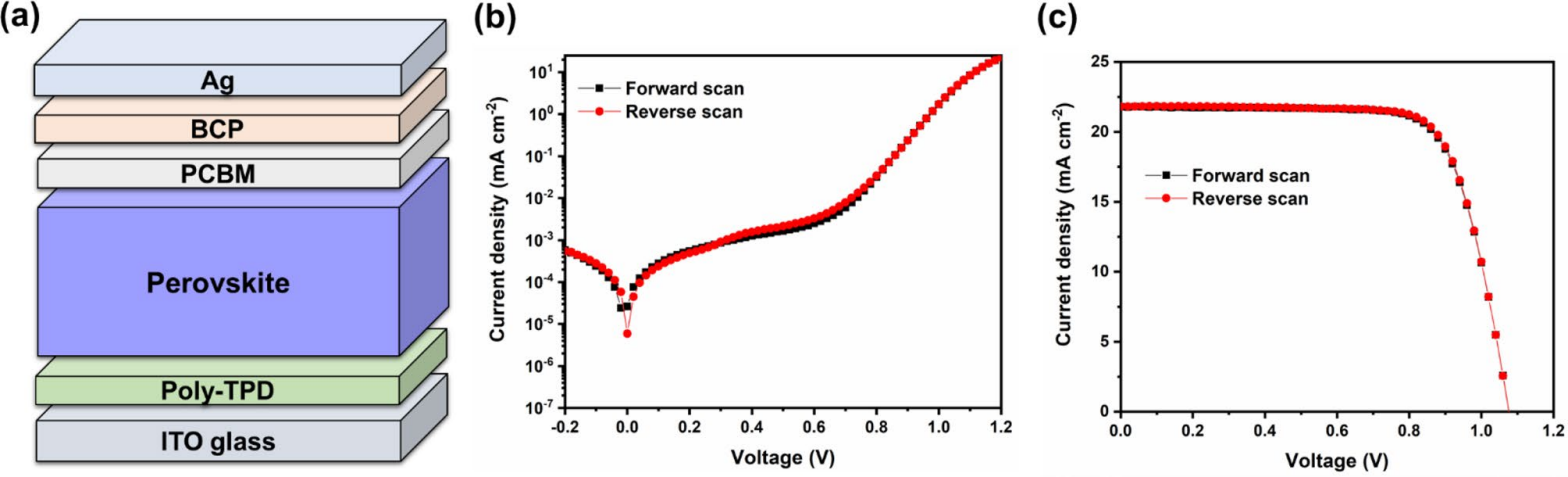
Inverted PSCs based on the slot-die perovskite film: (**a**) schematic device configuration; (**b**) *J*–V curves in the dark; and (**c**) *J*–V curves at one-sun illumination. *Note*: the *J*–V curves represent the behaviour of a champion device.

To verify the superior quality of the deposited perovskite film through our developed slot-die coating process for preparing sample 4, we incorporated the MAPbI_3_ thin-film into inverted (*p-i-n*) PSCs with a structure of Glass/ITO/poly[*N*,*N*′-bis(4-butylphenyl)-*N*,*N*′-bis(phenyl)benzidine] (poly-TPD)/perovskite/[6, 6]-phenyl-C61-butyric acid methyl ester (PCBM)/bathocuproine (BCP)/Ag. As shown in Fig. 6a, the perovskite is sandwiched by the poly-TPD as the hole transport layer and PCBM as the electron transport layer, both of which contributed to the excellent performance in the inverted PSCs^61,62^. In poly-TPD, *p*-type fluorinated F4TCNQ is also added to increase the open-circuit voltage (*V*_*OC*_). The semi-log dark *J*–*V* curves in Fig. 6b show typical diode characteristics and the plot shape can be divided into four different regions. From low to high voltage, the physical processes depicted by the semi-log *J*–*V* curve are attributed to the shunt resistance (*R*_*sh*_), recombination, diffusion, and the series resistance (*R*_*s*_) of the device^63^. The reverse saturation current densities (*J*_o_) can be extracted by the following equitation:

4 $$\mathrm{ln}\left[J-\frac{\left(V-JA{R}_{s}\right)}{A{R}_{sh}}\right]=\mathrm{ln}{J}_{0}+\frac{q}{nkT}\left(V-JA{R}_{s}\right)$$

wherein *q, k, n, T, A* are the elementary charge, Boltzmann constant, ideality factor*,* absolute temperature, and device area^64^. Considering almost identical forward and reverse scans at the second exponential region, we linearly fitted the reverse curve close to the diode turn-on voltage as shown in Suppl Fig. S5)^65^. The intercept is -19.6 and the *J*_o_ was calculated to be 3.1 × 10^–9^ mA cm^−2^, indicating a low current leakage and carrier recombination in the whole device. This is attributed to high quality of the perovskite film, and low defect and carrier recombination at the interfaces between perovskite film and charge selective layers inside the device. As shown in Fig. 6c, at one-sun illumination the fabricated devices display a champion efficiency of over a 17.5% with a slightly lower average value of 12.6 ± 2.1%, as summarized in Table 2. We ascribe this efficiency decrease to the small substrate size used (the slot-die coating method does not allow for the deposition of homogenous layers over very short distances). Nevertheless, the achieved efficiencies are comparable with results reported for slot-die coated PSC devices fabricated from ACN-based inks^46,48,49,66^. Furthermore, all devices show negligible hysteresis in their current density–voltage (*J*–*V*) curves (both in dark and one-sun illumination, Fig. 6b,c).

**Table 2:** Device performance based on slot-die coated perovskite films with the architecture Glass/ITO/poly-TPD/perovskite/PCBM/BCP/Ag.

Device	Scan direction	*V*_*OC*_ (V)	*J*_*SC*_ (mA cm^-2^)	FF	PCE (%)
Champion	FS	1.08	21.8	0.74	17.4
Champion	RS	1.08	21.8	0.75	17.5
Average	FS	1.05 ± 0.02	17.5 ± 2.2	0.66 ± 0.07	12.2 ± 2.4
	RS	1.05 ± 0.02	17.3 ± 2.0	0.69 ± 0.05	12.6 ± 2.1

The average data is based on 15 single devices.

## Conclusion

In this work, perovskite-based precursor inks tailored for a one-step deposition of perovskite films at room temperature using the scalable slot-die coating technique were developed. The presented perovskite inks and coating method allow for the reproducible deposition of high quality MAPbI_3_ perovskite thin films, which showed full coverage over the deposited area. Various techniques for triggering the nucleation step and crystal growth inside the wet perovskite film were explored. It was found that an elevated substrate temperature during the coating process of precursor inks based on low-boiling point primary solvents, such as acetonitrile, negatively impacts the film formation process. This was related to the CGD-type formation mechanism in the perovskite film, which results in large grain clusters formation inside the dry film. On the other hand, room temperature slot-die coating of MAPbI_3_ inks, accompanied with an inert gas blowing can effectively enhance the perovskite film quality by changing the film formation mechanism from CGD- to ND-type, creating densely packed crystal grains in the film. The superior quality of the perovskite film achieved through the developed recipe was analysed and studied in this work. Using imaging techniques, such as SEM and AFM, we confirmed that a smooth MAPbI_3_ thin film can be deposited over large areas, offering densely packed micrometer size grains. PL and TRPL spectroscopy demonstrated that the developed deposition method can deliver a low level of recombination sites, and therefore defect density inside the perovskite film. The XRD results also confirmed the high crystallinity and purity of the prepared MAPbI_3_ layer, with an enhancement in the preferred crystal orientation in the <110> direction. It was also demonstrated that the proposed recipe for perovskite film deposition could improve the optoelectronic characteristics of the final film. As a result, PSC efficiencies exceeding 17.5% were achieved. Overall, the developed perovskite precursor inks and the tailored slot-die coating deposition technique offers a cost-effective manufacturing of high-quality perovskite films, suitable for various applications, through a simple, reliable, and reproducible method.

## Supplementary Information

Supplementary Information.
