# Supplementary material for: A route towards the fabrication of large-scale and high-quality perovskite films for optoelectronic devices
Source: Sci Rep. 2022 May 6;12:7411. doi: 10.1038/s41598-022-10790-z (PMC9076914; doi:10.1038/s41598-022-10790-z)
Supplement: Supplementary file 1 — Supplementary Information. [file 41598_2022_10790_MOESM1_ESM.docx]

**Supplementary Information**

**A route towards the fabrication of large-scale and high-quality perovskite films for optoelectronic devices**

**Ehsan Rezaee^1^, Dimitar Kutsarov^1^, Bowei Li^1^, Jinxin Bi^1^, S. Ravi P. Silva^1^***

^1^ Department of Electrical and Electronic Engineering, Advanced Technology Institute (ATI), University of Surrey, Guildford, Surrey GU2 7XH, UK

E-mail: s.silva@surrey.ac.uk


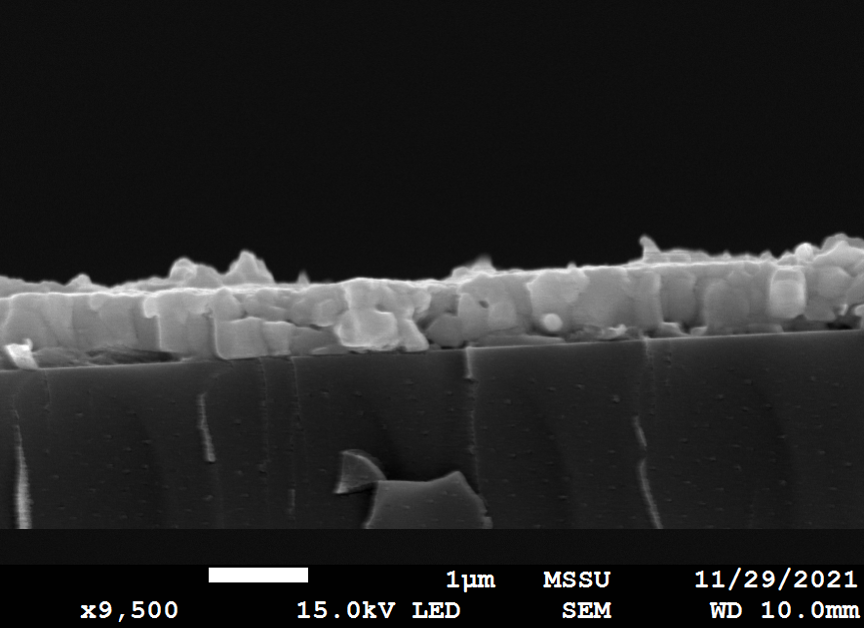


**Figure S1.** Cross-section SEM image of sample 4.


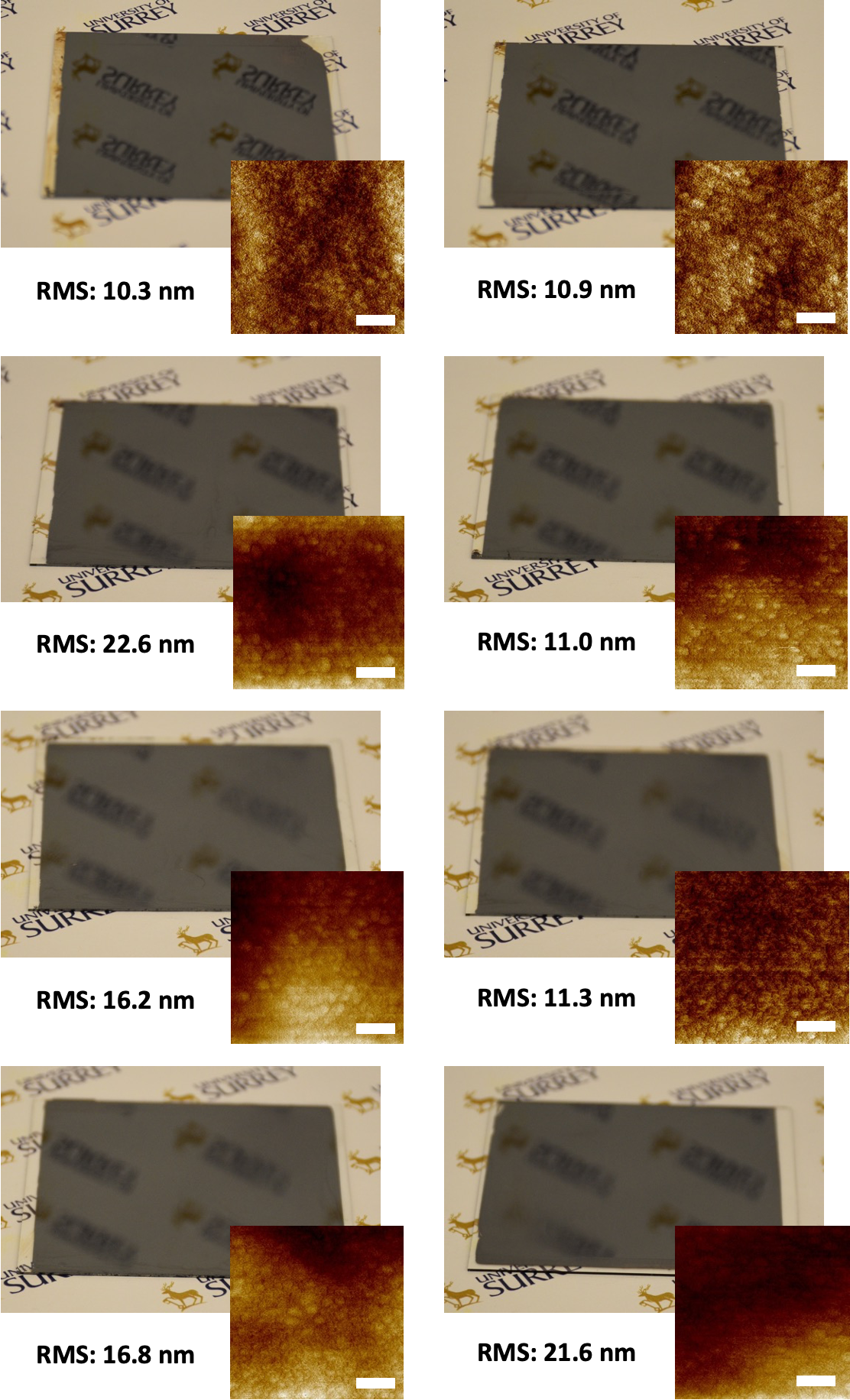


**Figure S2.** Deposition reproducibility of the developed ink for sample 4: eight samples were prepared, delivering the same high quality and coverage for the perovskite film. The bars in the AFM images are 1 μm length.


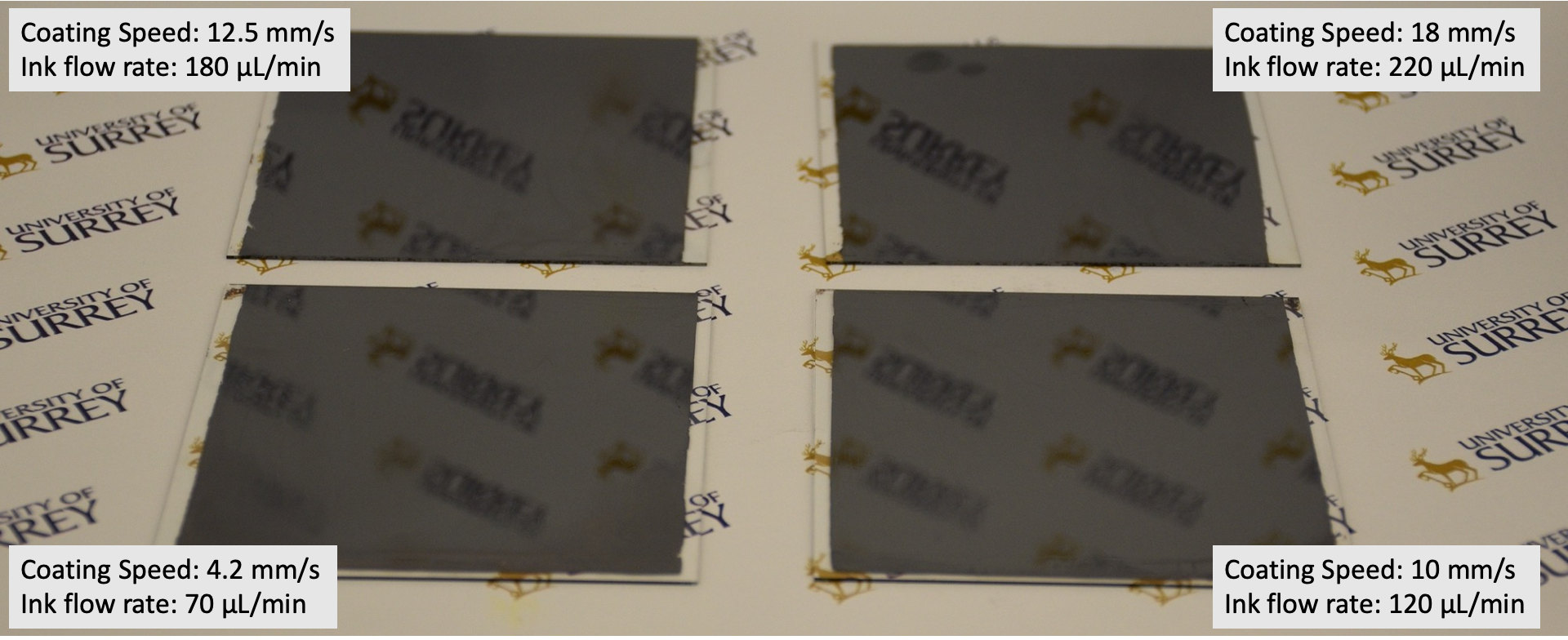


**Figure S3.** Quality of the deposited perovskite films depending on the coating speed: the film coverage and quality is stable regardless of the coating speed increase from 4.2 mm/s to 18 mm/s. Note: the ink flow rate was increased accordingly to the increased coating speed to match the required ink supply.


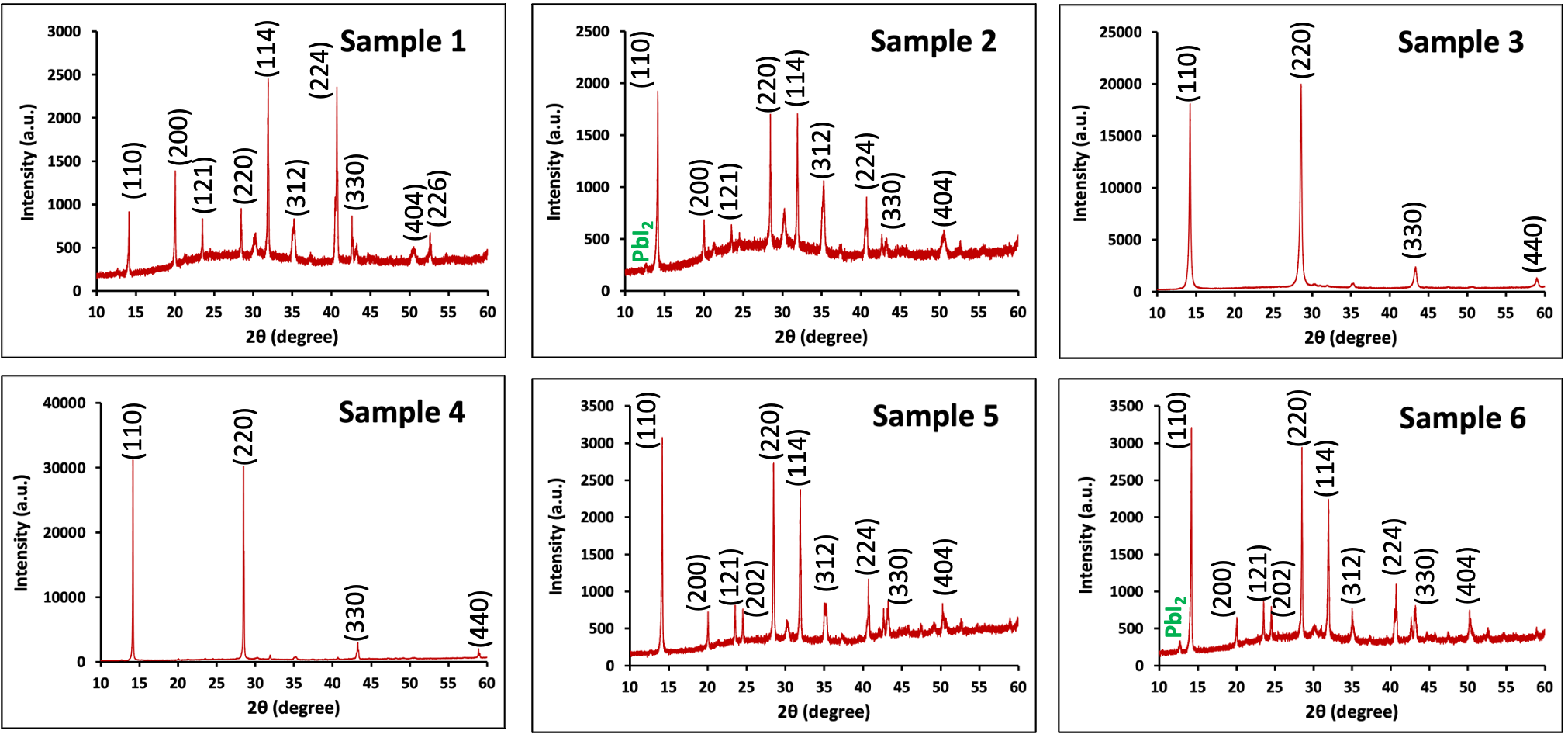


**Figure S4.** XRD spectra of the prepared thin films for samples 1-6.


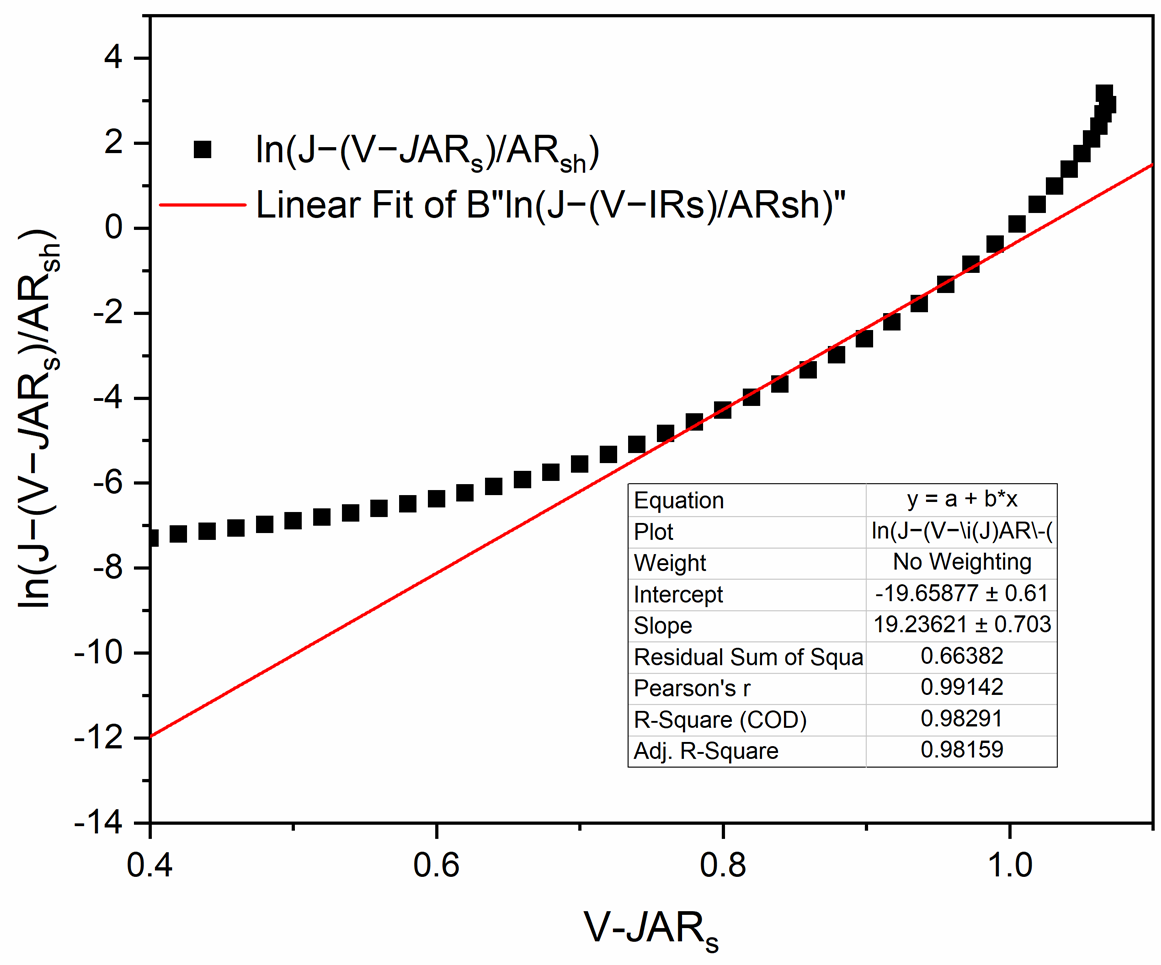


**Figure S5.** Linear fit and calculation of *J*0 from the dark *J-V* curves of the champion device.

**Table S1.** Fitted parameters of TRPL spectra for samples 1-6.

| **Sample code** | **A_1_** | **τ_1_(ns)** | **A_2_** | **τ_2_(ns)** | **τ_avg_(ns)** |
| --- | --- | --- | --- | --- | --- |
| **Sample 1** | 2.770 | 44.5 | 5.057 | 11.1 | 34.05 |
| **Sample 2** | 2.734 | 30.17 | 6.400 | 7.36 | 21.87 |
| **Sample 3** | 2.249 | 38.25 | 5.770 | 4.76 | 30.14 |
| **Sample 4** | 3.660 | 121.7 | 4.660 | 63.6 | 98.49 |
| **Sample 5** | 3.770 | 50.63 | 6.380 | 19.25 | 38.36 |
| **Sample 6** | 2.404 | 46.66 | 5.710 | 1.56 | 43.34 |
